# An effective robot selection and recharge scheduling approach for improving robotic networks performance

**DOI:** 10.1038/s41598-024-78747-y

**Published:** 2024-11-18

**Authors:** Shimaa E. ElSayyad, Ahmed I. Saleh, Hesham A. Ali, M. S. Saraya, Asmaa H. Rabie, Mohamed M. Abdelsalam

**Affiliations:** 1 Computers and Control Systems Engineering Department Faculty of Engineering, Mansoura University, Mansoura, 35516 Egypt; 2 Misr Higher Institute for Engineering and Technology, Mansoura, Egypt; 3 Faculty of Artificial Intelligence, Delta University for Science and Technology, Gamasa, Mansoura, Egypt; 4 Faculty of Engineering, Mansoura National University, Mansoura, Gamasa Egypt

**Keywords:** Robotics, Path planning, Cloud Computing, Fog, Fuzzy logic, Ranking, Electrical and electronic engineering, Computational models, Computational platforms and environments, Data mining, Data processing, Databases, Hardware and infrastructure, Machine learning, Network topology, Probabilistic data networks, Software, Information theory and computation, Techniques and instrumentation, Energy grids and networks

## Abstract

With the ability of servers to remotely control and manage a mobile robot, mobile robots are becoming more widespread as a form of remote communication and human-robot interaction. Controlling these robots, however, can be challenging because of their power consumption, delays, or the challenge of selecting the right robot for a certain task. This paper introduces a novel methodology for enhancing the efficacy of a mobile robotic network. The key two contributions of our suggested methodology are: I: A recommended strategy that eliminates the unwieldy robots before selecting the ideal robot to satisfy the task. II: A suggested procedure that uses a fuzzy algorithm to schedule the robots that need to be recharged. Since multiple robots may need to be recharged at once, this process aims to manage and control the recharging of robots in order to avoid conflicts or crowding. The suggested approach aims to preserve the charging capacity, physical resources (e.g. Hardware components), and battery life of the robots by loading the application onto a remote server node instead of individual robots. Furthermore, our solution makes use of fog servers to speed up data transfers between smart devices and the cloud, it is also used to move processing from remote cloud servers closer to the robots, improving on-site access to location-based services and real-time interaction. Simulation results showed that, our method achieved a 2.4% improvement in average accuracy and a 2.2% enhancement in average power usage over the most recent methods in the same comparable settings.

## Introduction

Mobile robots can work alongside people in most occupations or even may be replaced^1^. For example: Manufacturers’ labour costs are undoubtedly reduced by the increasing integration of robots into the majority of industrial sectors and their daily life^2^. Additionally, the application of mobile robotics in emergency scenarios in homes, hospitals, and healthcare facilities^3,4^. Path planning, or the process of finding a collision-free path between a beginning and an ending position inside a workplace, is one of the main tasks carried out by mobile robotic networks. The path may be optimized and the algorithm’s performance assessed using a variety of measures, such as the algorithm’s processing time, travel time, power usage, etc. Path planning requires the start and destination locations, a good mapping of the workspace, determining which robot is best suited to complete a task, and preserving battery life^5^.

The Fog platform^6^employs processing power from distant cloud servers to be closer to end users’ locations to improve their access to location-based services and instant engagement. The amount of data stored on cloud servers is significantly decreased by the Fog’s on-site processing capability. The cloud and end-user device levels are separated by the edge network layer. The cloud is provided with routing, real-time communication, and computational proxies. The study published in^7^ proposes a novel deep learning-based technique wherein the authors integrate a recurrent neural network (RNN) and the reinforcement learning algorithm to predict the teleoperator’s actions and balance the latency of cloud interactions.

Our study concentrates on two key issues that impact mobile robot navigation inside a workspace: firstly, we have to select the best robot for each activity to speed up delivery, and secondly, the robot must conserve its battery life by scheduling recharge. As a result, the primary goal and contribution of the suggested framework is to enhance the selection of robots for a given task by evaluating the robots that are present in a certain workspace. Furthermore, our suggested standards combined with the robot’s power consumption guarantee that no robot would turn off due to a battery drain problem. One of the most important advantages of the suggested strategy is the quick response time, which is ensured by the lower latency that centralised systems may provide in comparison to dispersed ones.

This paper introduces a new approach that enhances the performance of a robot’s path planning. Our methodology has the following main contributions:

(I) The Selection Process (SP), which gathers data from various databases stored on Fog servers about the workspace and its resident robots. The fog server can select the robot that is best convenient to fulfil the request after removing robots that are inconvenient for completing the task. The request is a query for a robot to perform a specific task.
(II) The Recharge Schedule Process (RSP), a suggested procedure, uses a fuzzy algorithm to schedule the robots that need to be recharged. Since multiple robots may need to be recharged at the same time, this process aims to manage and control the recharging of robots to avoid conflicts or crowding.

The following is the structure of the paper’s roadmap: Sect. 1 presents the goals of the study, and a literature on recent developments in the subject of robot path-planning strategies. Section 2 discusses the suggested method and, system’s framework, and the procedures of the applied methodology a detailed discussion of the techniques and the suggested system is in Sect. 3. The testing and final findings evaluating the suggested approach, metrics for simulation analysis, benchmarks for efficiency, and an evaluation of our method’s effectiveness in comparison to existing methods are presented in Sect. 4. The last section concludes the suggested methodology and offers recommendations for further study.

Path planning problems were investigated using a variety of methods, including the following: Grid-Based Approaches, such as the A* and D* methods in^8^, Window Approaches, such as DWA method introduced in^9^;; Potential Field Method, such as APF introduced in^10^; Sampling-Based Planning method presented in^11^, Reinforcement Learning technique used in^12^; and intelligent-based algorithms utilized and developed in^13–15^.

Due to its accuracy, maximum efficiency, and optimal efficiency, the graph traversing and pathfinding algorithm A* calculates the shortest route (about the provided values) from start to destination given a weighted network, a source node, and a target node^16^. Numerous fields have profited from the advancement and personalisation of artificial intelligence, such as robotic route planning, automated control, graph theory, intelligent urban planning, and gaming^17,18^. It computes the outcome of the function known as heuristic for every node on the workspace and looks at numerous nearby nodes to identify the optimal solution with a zero probability of collision. The A* methodology is a popular path-planning method for graph traversal. By dividing an issue into smaller parts or phases, A* provides a step-by-step solution to a specific problem^19^, making it easier to convert an issue into a program. Because the method requires a certain amount of time, static settings are preferred over dynamic ones.

The D* (or Dynamic A*) method^20^ is used to create a path free of collisions between moving obstacles. Both the cost map and the previously created one are partially repaired using the accurate incremental search method D*. A robot’s status is processed using the D* algorithm until it is removed from the open list. The current state sequence and its reverse pointers are calculated simultaneously to get the robot closer to the goal or to modify the cost owing to obstacles that was previously identified and add the pertinent state to the open list. A new cost function computation is necessary for the D* algorithm. Because of its large overhead, cost re-computation in the Modified D* technique only happens when absolutely necessary.

Rapidly-Exploring Random Trees (RRT)^21^ is an online, dynamic algorithm that doesn’t follow a set course. Rather, they spread throughout the whole area and use the weights allotted to each node to build a path from point A to point B. RRTs were developed to address a range of path-planning issues. They were created especially to deal with limits that can’t be comprised of positional restrictions. Both RRTs and Probabilistic Road Maps (PRMs) have been generated with the same desired qualities, employing arbitrary parameters and minimum heuristics. Better performance and more consistency in the outcomes are the outcomes of this. RRTs do not require the development of any connections between states to solve a problem, in contrast to PRMs which may request interconnections across dozens of configurations or states. As an outcome, using RRTs for non-holonomic and dynamic planning is made simpler.

A team of academics created the Dynamic Window Approach (DWA)^22^as a method for mobile robots to prevent collisions online. The dynamic window technique, was specifically designed to manage the limitations given by the robot’s limited speeds and accelerations. It is produced instantaneously by the robot’s mechanisms. It is made up of two main parts: finding the optimal solution inside the defined search space and creating a precise search space. According to^23^ the search space consists of safe, fast-moving, collision-free circular pathways. The goal of optimization is to select an orientation and velocity that will minimize obstacles in the path of the robot as it travels to its destination. It is safe by design and has been shown to work fairly effectively in test installations.

For local path planning, the dynamic window method is a useful technique. However, because multiple of its evaluation functions appear insufficient and there is no method for figuring out their weights, it is dependent on the global context and has issues in a variety of circumstances. For local and global path planning, the Artificial Potential Field approach (APF)^24^ can be applied. Building an artificial field of potential with a goal and an impediment that each generates a gravitational field is the basic idea. The robot travels towards its target using the least amount of total repulsive force feasible. It then carries out this procedure as frequently as required to accomplish its goal. The potential field method for robot navigation works well in spite of the local minima issue. The robot comes to a stop when its net force becomes zero. Unless its surroundings alter, the robot will have no way to get there.

Regardless of whether the workspace is static or dynamic whether the map is configured on a local or global scale, recent path planning algorithms have proven useful in determining the best path for a robot to fulfil a given task in a specified work area. However, these algorithms struggle to strike a balance between speed and precision, which can affect how well they function.

## Method

**Fig. 1:**
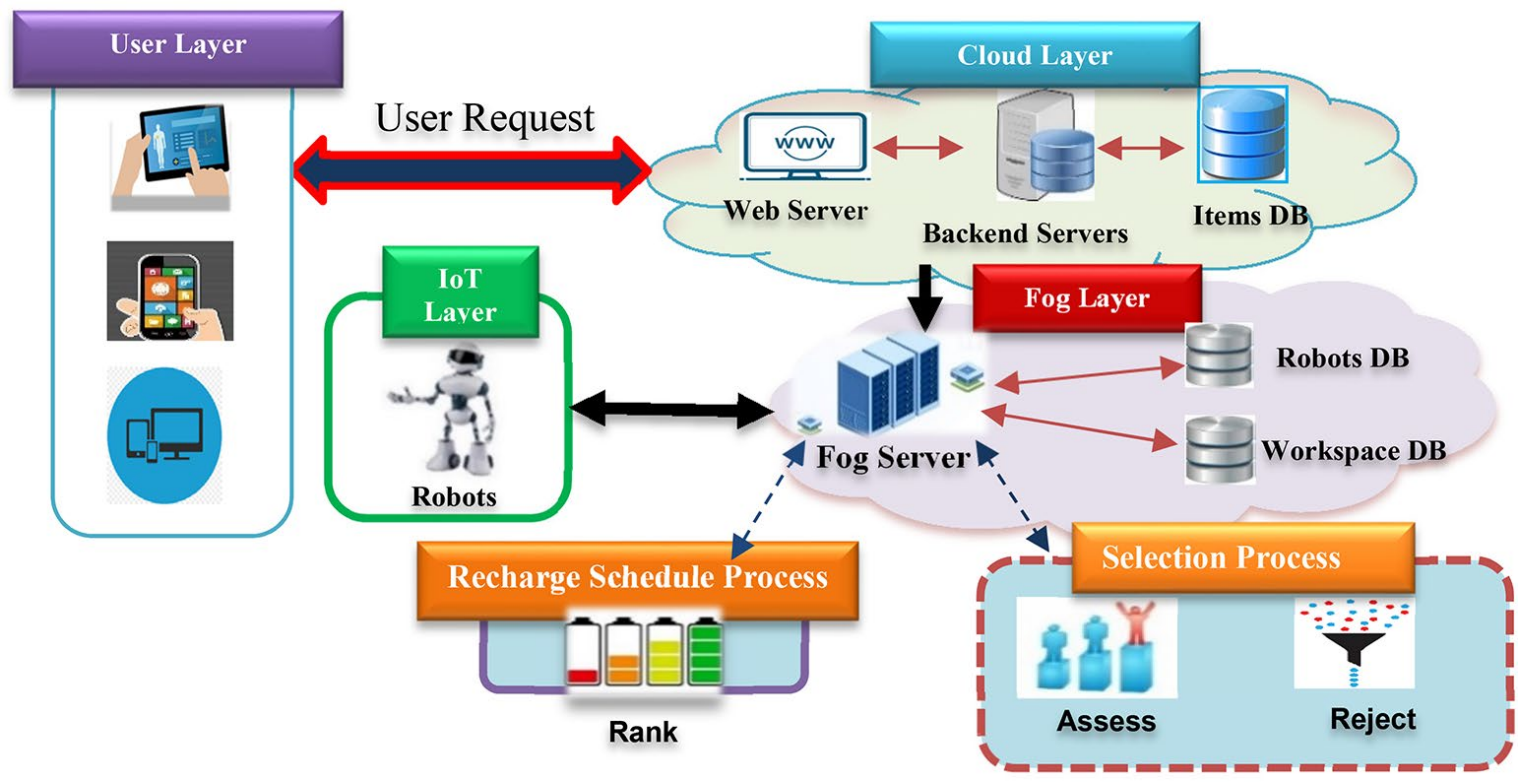
Main layers used for the implementation of the proposed approach.

The user layer, IoT layer, cloud layer, and fog layer are the four main layers of the suggested structure that are depicted in Fig. 1. I Cloud Layer: Portals that handle and store user requests, item databases, web servers, and cloud data centres are all included in this layer. (II) Fog Layer: To operate and manage the real-time robots, a set of servers is utilized to load the suggested selection and robot recharge scheduling processes. (III) IoT Layer: consists of a set of mobile robots that send signals to the fog servers with the required data (e.g., location) via sensors. (IV) User Layer: Through the cloud layer, end users interact with the system through apps housed in this layer.

By serving as a bridge between the cloud and IoT devices, the fog layer offers a distributed computing platform that is closer to the IoT layer. This has a number of benefits for real-world implementations, including decreased latency for real-time decision making, offloading large processes off the cloud, increased reliability, compliance with security regulations, achievement of data privacy, and increased solution efficiency. The connectivity between cloud and fog layers can vary depending on the network architectures and the application technical requirements, in our proposed system we used the most common connectivity type, which is “VPN Tunneling connectivity“either by IPsec or SSL/TLS are valid. In addition, the connectivity between the Fog and IoT devices uses the Wireless Signals^25^ (e.g., W-Fi, Bluetooth, and Cellular Networks). For messaging and communication between cloud and fog layers in IoT systems, various types of signals and protocols are involved, they are typically transmitted using a combination of protocols, like ROS (Robot Operating System), MQTT (Message Queuing Telemetry Transport): suitable for IoT applications, HTTP/HTTPS: For web-based communication and API calls, CoAP (Constrained Application Protocol): A specialized protocol designed for resource-constrained devices in IoT environments, WebSocket: For real-time, bidirectional communication between the cloud and fog, SSH: For secure remote access, SOAP: For web services, and REST: For web APIs.

**Fig. 2:**
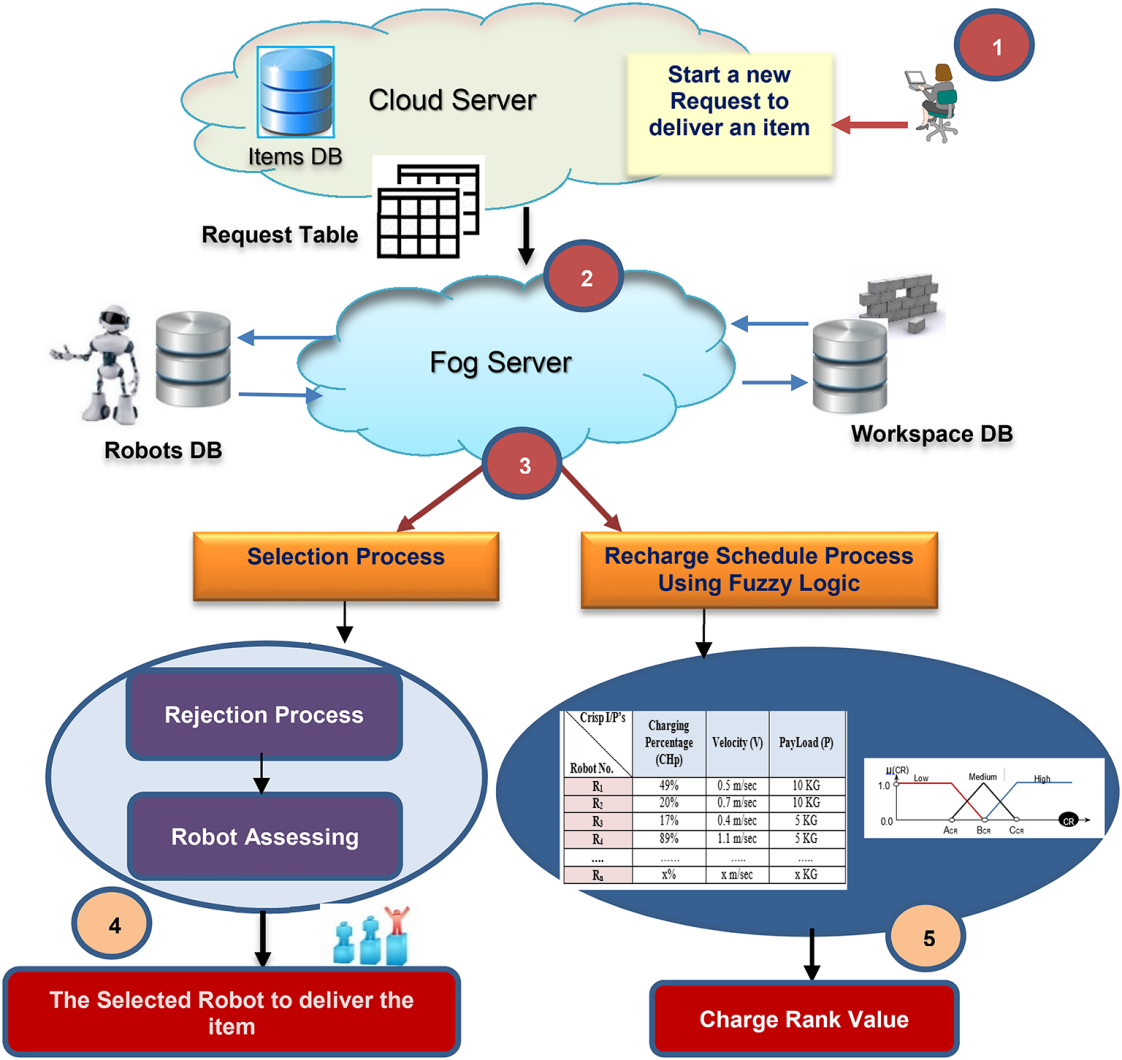
The Proposed approach workflow diagram.

The proposed framework as shown in Fig. 2 takes the following steps: first, a new request for an item takes place and the requested item table is sent through the cloud layer to a fog server; next, the proposed methodologies at the fog layer server selects the most appropriate robot to perform the task, and, offers a robot’s recharge schedule process that uses a fuzzy-based ranking algorithm to determine which robot needs to recharge by calculating a charge rank value. The two main processes in our technique are the Selection Process (SP) and the Recharge Schedule Process (RSP). The fog server loads the processes separately. While the Robots Database (RDB) on the fog server contains variant and static information for each robot residing in the workspace, the Workspace DB, on the Fog server, contains information about the workspace’s area (e.g., static blocks).

**Table 1:** Robots Database (RDB).

R_ID	Max_P	V	Position	CHp	CHt	Charge Schedule	Status	Updated time (From)	Event Trigger
R1	6 KG	0.5 m/sec	15,21	44%	19 min	No	Available	97 s	Item Delivered
R2	2 KG	0.7 m/sec	6,10	21%	5 min	Scheduled	Busy	14 s	Item Received
R3	5 KG	0.4 m/sec	33,3	80%	11 min	Charging	Charging	112 s	Charging Point
R4	10 KG	1.1 m/sec	11,60	89%	36 min	No	Available	264 s	Item Delivered
Rn	x KG	x m/sec	x, y	x%	X min	.	.	.	.

**Fig. 3:**
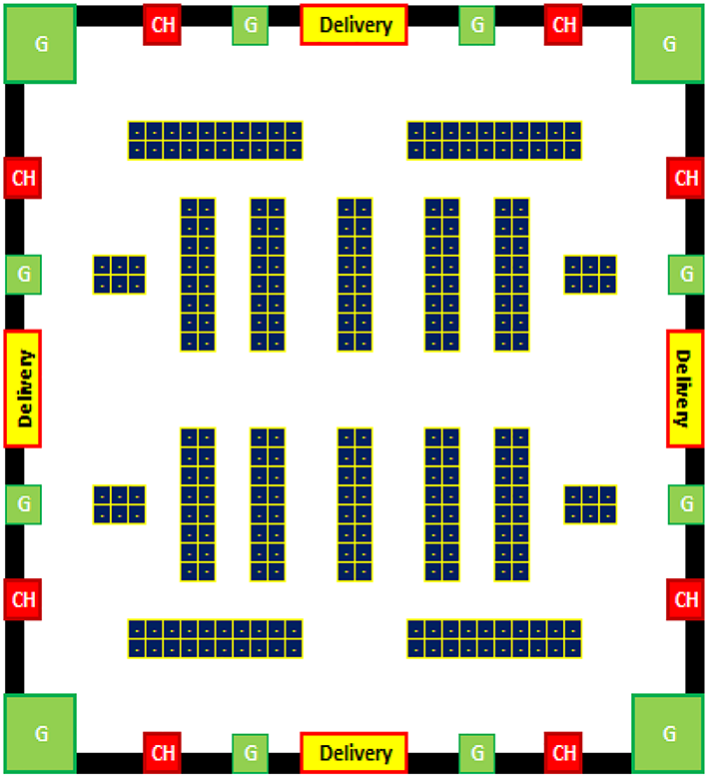
An example of workspace Binary Gridmap representation.

Two types of data are stored for every robot, as Table 1 shows. The first group includes static information, like: (I) R_ID: which is the Robot ID. (II) The highest weight that the robot may carry is called Max_Payload (Max_P). (III) Each robot’s speed in meters per second is expressed using the velocity (V). The second category of data contains dynamic details that are altered with each event with each robot. Dynamic data, such as position, which shows the robots location on the gridmap representation, as shown in Fig. 3. The robot’s charging percentage (CHp) at a given checkpoint. Charge Time (CHt) shows the remaining time to recharge the robot, which shows how long the battery’s life will last. The robot’s overall status (available, busy, or charging) is represented by Status, also its charging schedule status is represented in the table.

Every robot typically transmits messages to the fog server regularly. Due to the robot’s battery expiring quickly and the Fog servers’ memory being used up by recording numerous unimportant signals, this approach is tedious and inefficient. To address these problems in our methodology, we proposed that, in response to particular presumed events, every robot would send its dynamic information. Using this method the supervising server and the robots’ message protocols are established. The assumed events are as follows:

I. The robot begins to charge at CH (Charging nodes) as shown in Fig. 3.
II. Robot is completely charged.
III. A request has just sent to the robot by the fog server.
IV. Robot carried an item: a robot has just pick up an object to move it to the intended spot.
V. Robot reached target: the moment the robot reached the destination and delivered the item.
VI. Robot hibernating at G (Green) zones which are the locations in which the robot could be in a hibernating mood when there is no task to be done to save power as shown in Fig. 3.
VII. The robot reached the minimal power threshold.

## The proposed approach

The suggested method consists of two primary processes: the selection process, which chooses the robot most suitable to fulfil the request, and recharge scheduling task, which uses a fuzzy logic algorithm to manage and organise the process of robot recharging.

### Selection process (SP)

This process determines which robot is most suitable for a given task by looking at both the robot’s capabilities and the demands of the request. The three main components of SP are the Requests Table (RT), Rejection Process (RP), and Robot Assessing (RA).

**Table 2:** Request table (RT).

User	R_Priority	Item Code	Server Area	Weight	Source Position	Target Position
User 80	Low	Item C-1	Fog A	0.5 KG	120,88	100,30
User 60	Critical	Item A-123	Fog B	7.3 KG	22,10	10,0
User 21	Normal	Item C-72	Fog B	1.9 KG	97,60	130,140
User 114	Normal	Item B-12	Fog C	9 KG	100,30	50,70
User 9	Low	Item A-45	Fog A	2.5 KG	87,23	0,40
User 52	Critical	Item B-172	Fog B	4.3 KG	10,5	23,90

After receiving user requests, the fog server compiles all pertinent information for every query in a table called the request Table (RT). Table 2 shows an example of RT tables related to a particular event. All the item-related data is stored on the cloud server, where R_Priority is the Request Priority.

The Rejection Process (RP) is a proposed method for the second step of selection that takes into account certain presumption criteria in order to reduce the number of robots that can be selected to finish a task and fulfil a request. As a result, the server needs less time to process requests. Algorithm 1 shows the algorithm that was used to decide which robots would be rejected and which should be chosen to complete a task. For a robot to be rejected, a number of putative robot-related requirements need to be fulfilled. The algorithm searches the RDB to determine the robot’s start status; if the robot charges at the moment, it will be rejected. On the other hand, if a robot has started to work on an activity (such delivering an item), the algorithm uses the Requests Table to decide the request priority. Either the request status is low or normal, the robot will be refused.

**Algorithm 1:**
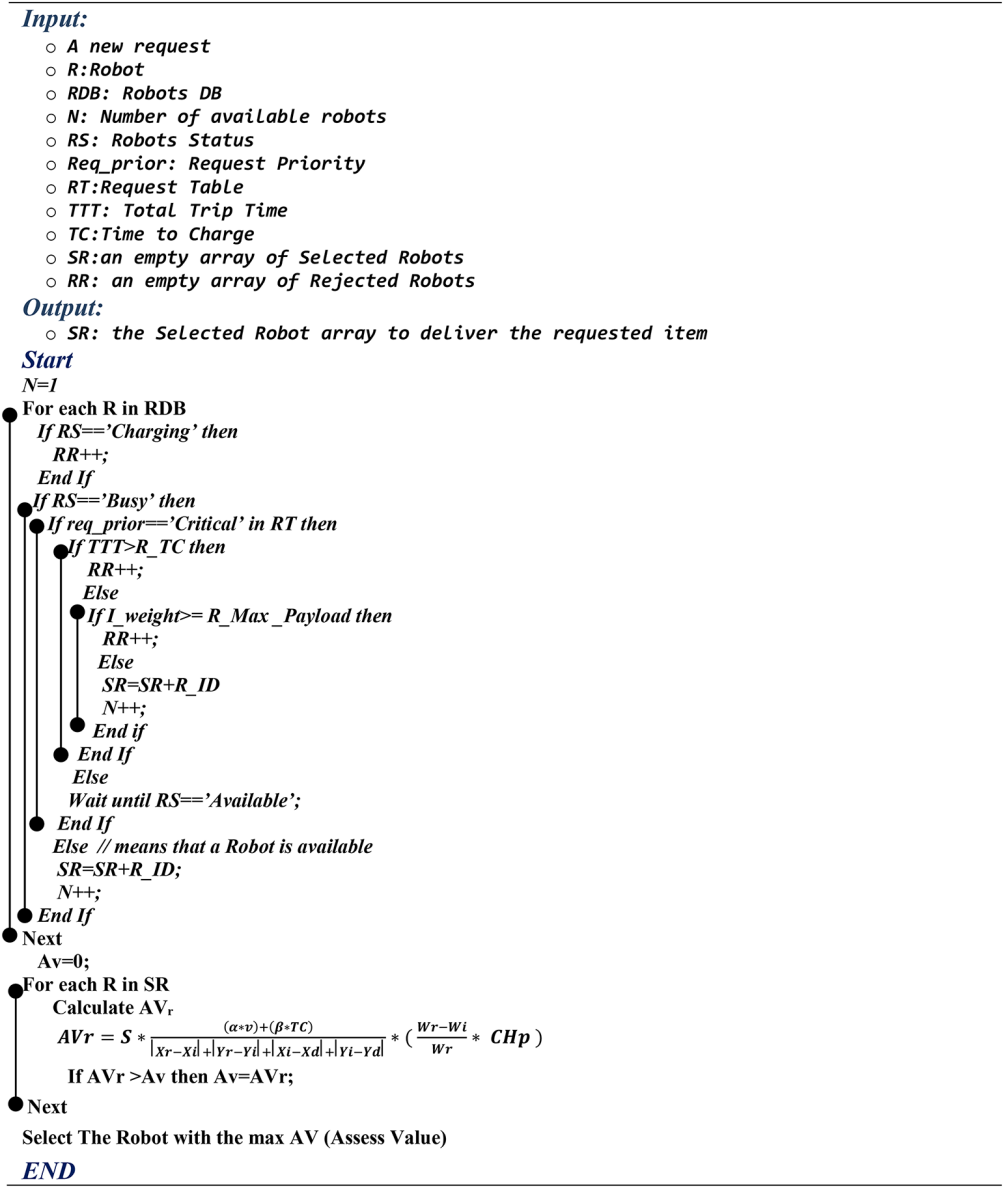
.

However, a robot’s remaining charging time (TC) will be taken into account when request priority is crucial. The robot is going to be refused if the item time for delivery exceeds the Total Travel Time (TTT), which is the amount of time that is necessary for the robot to travel from the starting point to the destination. Furthermore, the overall weight of the requested item, which is obtained through the Requests Table (RT), is considered. If the weight of the ordered item is above the robot’s payload limit, the robot is declined; if not, it is selected to participate in the Robot Assessing (RA) phase. Once undesired robots have been eliminated, RA, according to the proposed formula, gives an assessing value (AV) for every robot in the set of robots chosen by the previous selection process to process an incoming request. Equation 1 shows the conventional procedure for giving each robot an Assessing Value.

1 $$\:AV=\varvec{S}*\frac{\left(\alpha\:*v\right)+\left(\beta\:*TC\right)}{\left|Xr-Xi\right|+\left|Yr-Yi\right|+\left|Xi-Xd\right|+\left|Yi-Yd\right|}*\left(\:\frac{Wr-Wi}{Wr}*\:CHp\:\right)\:\:\:\:$$

Where: S represents the robot’s status, which equals zero when it is charging or performing a task as specified in RDB and one if the robot is available, and AV stands for the Assess value. The variable v represents the robot’s velocity value, while TC denotes the approximate remaining time for charging the robot. The coordinates of the destination are (Xd, Yd), the coordinates of the object’s current location are (Xi, Yi), and the coordinates of the robot’s current location are (Xr, Yr).

The power factors for v and TC are denoted by α and β, respectively. The actual weight of the item and the current robot charge percentage, as established by the RT table, are denoted by the acronyms Wi and CHp, respectively. The maximum Payload threshold for the robot is Wr, and it is derived from the RDB. Algorithm 1 illustrates the procedures of selecting the most convenient robot using the Assess Value.

### Recharge scheduling process (RSP)

Fuzzy logic^26^is used in recharge scheduling to rank and then identify robots that require recharging based on three primary features: Charging Percentage (CHp), Velocity (V), and PayLoad (P). Accordingly, RSP can assign a Charging Rank (CR) to each robot using fuzzy inference system by considering its three pre-defined features (CHp, V, and P). In fact, fuzzy inference system is an approximate or uncertain reasoning system that can accurately make a decision based on incomplete information. In fact, there are four common membership functions called triangular, trapezoidal, gaussian, and sigmoidal^26^. The choice of membership function depends on the specific application and the nature of the fuzzy set. In this work, fuzzy inference system will be implemented on the values of CHp, V, and P features to provide CR for each robot based on trapezoidal function as shown in Table 3.

**Fig. 4:**
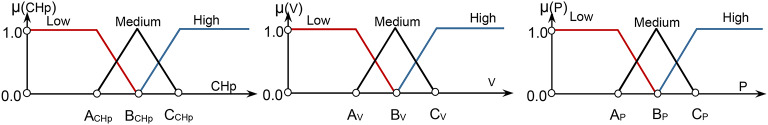
The considered three input membership functions for the three fuzzy sets.

Three grades of membership called ‘‘Small’’, ‘‘Medium’’, and ‘‘Large’’ are used to define the linguistic variables of fuzzy system for converting crisp inputs into these grades as shown in Fig. 4. The mathematical representation of membership functions for the small, medium, and large fuzzy sets is provided in Eqs. (2)–(4):

**Table 3:** Crisp inputs of the fuzzy inference system.

Robot No.	Crisp I/*P*’s		
	Charging Percentage (CHp)	Velocity (V)	PayLoad (*P*)
R_1_	49%	0.5 m/sec	10 KG
R_2_	20%	0.7 m/sec	10 KG
R_3_	17%	0.4 m/sec	5 KG
R_4_	89%	1.1 m/sec	5 KG
….	……	….	….
R_n_	x%	x m/sec	x KG

2 $$\:{\mu\:\left(y\right)}_{small}=\left\{\begin{array}{c}1\:\:\:\:\:\:\:\:\:\:\:\:\:\:\:\:\:\:y\preccurlyeq\:A\\\:\frac{B-y}{B-A}\:\:\:\:\:\:\:\:\:\:A\prec\:y\preccurlyeq\:B\\\:0\:\:\:\:\:\:\:\:\:\:\:\:\:\:\:\:\:\:\:y\succ\:B\end{array}\right.$$

3 $$\:{\mu\:\left(y\right)}_{medium}=\left\{\begin{array}{c}0\:\:\:\:\:\:\:\:\:\:\:\:\:\:\:\:\:\:y\preccurlyeq\:A\\\:\frac{y-A}{B-A}\:\:\:\:\:\:\:\:\:\:\:\:\:A\prec\:y\preccurlyeq\:B\\\:\frac{C-y}{C-B}\:\:\:\:\:\:\:\:\:\:\:\:\:B\prec\:y\preccurlyeq\:C\\\:0\:\:\:\:\:\:\:\:\:\:\:\:\:\:\:\:\:\:\:y\succ\:C\end{array}\right.$$

4 $$\:{\mu\:\left(y\right)}_{large}=\left\{\begin{array}{c}0\:\:\:\:\:\:\:\:\:\:\:\:\:\:\:\:\:\:y\preccurlyeq\:B\\\:\frac{y-B}{C-B}\:\:\:\:\:\:\:\:\:\:B\prec\:y\preccurlyeq\:C\\\:1\:\:\:\:\:\:\:\:\:\:\:\:\:\:\:\:\:\:\:y\succ\:C\:\end{array}\right.$$

The values of A, B, and C for each fuzzy input illustrated in Fig. 4a based on Table 3 are calculated using Eqs. (5)–(7)^23^:

5 $$\:{A}_{i/p}=\frac{0.5\text{*}\sum\:_{i=1}^{n}{\left(value\:\right)}_{i}}{n}$$

6 $$\:{B}_{i/p}=2\text{*}{A}_{i/p}$$

7 $$\:{C}_{i/p}=3\text{*}{A}_{i/p}$$

Where the number of input values is n and the value of ith robot at specific input feature is value. Then, the input of the fuzzy rule base is the output of fuzzification process. Here, a set of rules are represented in the form; if (Q is W) AND (S is R). . THEN (U is X), where Q, S, and U are input variables (e.g., CHp, V, and P) and W, R, and X are the corresponding linguistic variables (e.g., low, medium, and high). Based on the used three memberships and the three input features, there are different 27 rules which are represented in Table 4.

According to Table 4, ‘S’ is ‘‘Small’’, ‘M’ is ‘‘Medium’’, and ‘L’ is ‘‘Large’’. To illustrate the idea, the fourth rule in Table 4refers to; if (CP is H AND V is M AND W is H ) THEN (Output is H). In fact, fuzzy rules inference depends on one of four different methods called drastic product, max–min, sum-dot method, and max-product inference^23^. During this paper, the max-min method is used as it depends on choosing a min operator for the conjunction in the premise of the rule as well as for the implication function and the max operator for the aggregation. Consider a simple case of two items of evidence per rule, the corresponding rules will be.


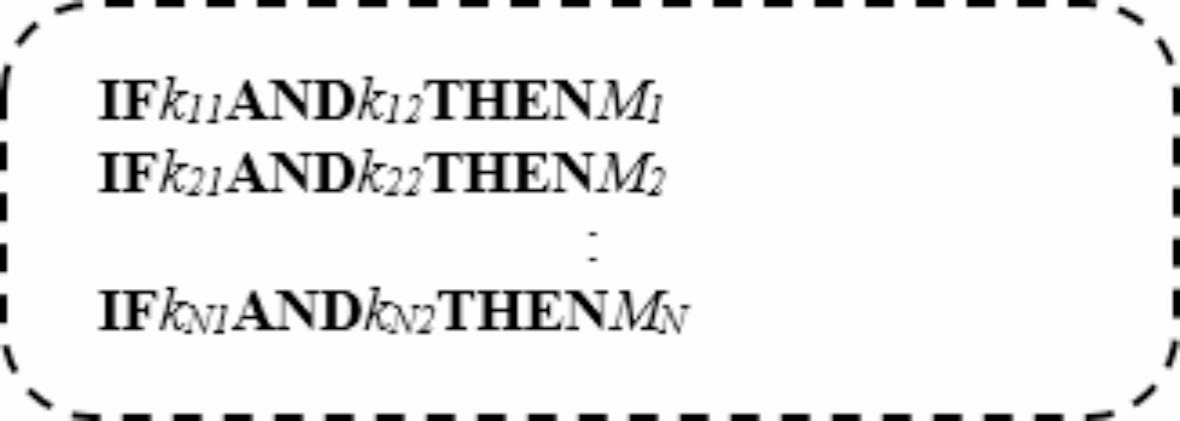


Therefore, the max-min compositional inference rule is:

$$\:\mu \:_{M} = \overbrace {{max}}^{{aggregation}}\left[ {\underbrace {{min}}_{{implication}}\left( {\mu \:_{{K_{{j1}} }} ,\mu \:_{{K_{{j2}} }} } \right)\:\forall \:\:j \in \:\{ {\text{1,2}},3, \ldots \:.,N\} } \right]$$

This yield;

$$\:{\mu\:}_{M}=max\left[min\left({\mu\:}_{{K}_{11}},{\mu\:}_{{K}_{12}}\right),\:min\left({\mu\:}_{{K}_{21}},{\mu\:}_{{K}_{22}}\right),\:\dots\:\dots\:.,\:min\left({\mu\:}_{{K}_{N1}},{\mu\:}_{{K}_{N2}}\right)\right]$$

The fuzzy rules will be defuzzified to give the output in a crisp form after inputs are fuzzified and then fuzzy rules are inferred from inputs. Hence, the defuzzification interface should be used to convert the output from fuzzy space into non-fuzzy (crisp) space because real time applications require crisp values. There are three main defuzzification methods, which are called; the mean of maxima, center-of-gravity, and maxcriterion^23^.

**Fig. 5:**
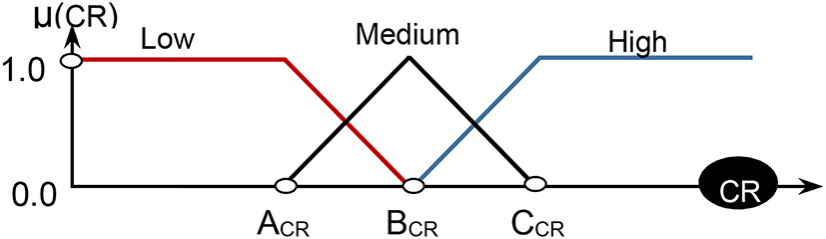
The output membership function.

**Fig. 6:**
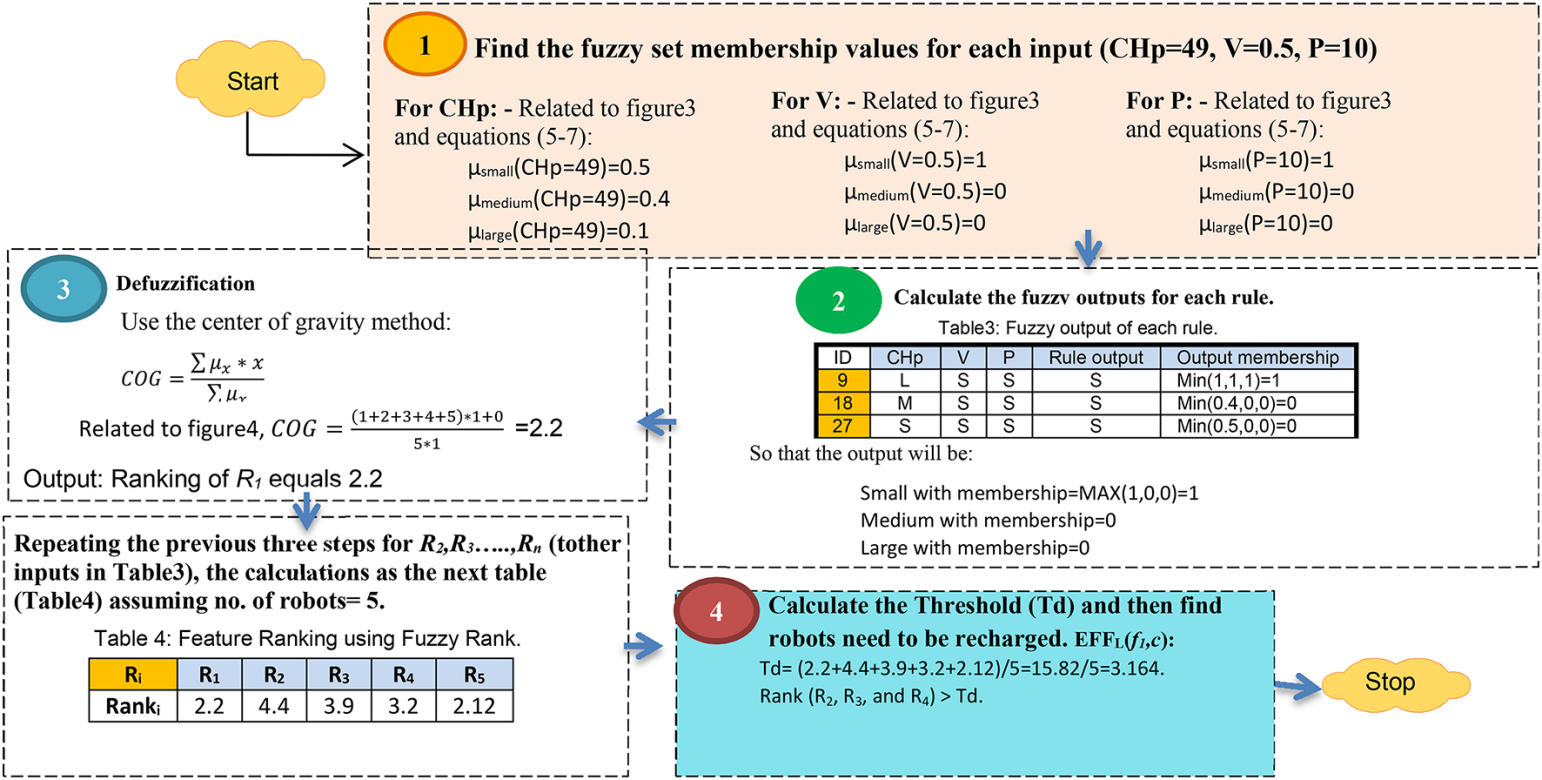
The steps of fuzzy inference system.

The most popular defuzzification method that is widely used in real applications is the Center of Gravity (CoG) method. CoG is similar to the formula of measuring the center of gravity in physics. Defuzzification can be constructed using the output membership function as illustrated in Fig. 5. Assuming ACR = 3, then BCR = 6, and CCR = 9 related to Eqs. (6) and (7). For illustrate the idea, fuzzy inference system is implemented on the three features presented in Table 3 as shown in Fig. 6. After calculating the ranks for robots, a Threshold (Td) value is used to determine which robots actually need to be recharged by using the average rank value using Eq. (8).

8 $$\:Td=\frac{\sum\:_{i=1}^{n}{Rank}_{i}}{n}$$

Where *n* is robots number and Rank is the rank value of *i*^*th*^ robot. Finally, every robot with a rank value greater than *Td* will be recharged.

**Table 4:** The used 27 different fuzzy rules.

ID	CHp	V	*P*	Rule output	ID	CHp	V	*P*	Rule output	ID	CHp	V	*P*	Rule output
1	L	L	L	L	10	M	L	L	L	19	S	L	L	L
2	L	L	M	L	11	M	L	M	L	20	S	L	M	S
3	L	L	S	L	12	M	L	S	M	21	S	L	S	S
4	L	M	L	L	13	M	M	L	M	22	S	M	L	M
5	L	M	M	M	14	M	M	M	M	23	S	M	M	S
6	L	M	S	M	15	M	M	S	M	24	S	M	S	S
7	L	S	L	L	16	M	S	L	M	25	S	S	L	S
8	L	S	M	M	17	M	S	M	S	26	S	S	M	S
9	L	S	S	S	18	M	S	S	S	27	S	S	S	S

## Results and discussion

Using MATLAB for simulation, the suggested approach was investigated^27^. Numerous extremely advanced and useful mathematical tools, such as Simulink and the Navigation Toolbox, are available in MATLAB. Therefore, by using mathematical functions, the total time required to develop the simulator can be greatly reduced. In addition, the object-oriented programming environment and robust built-in mathematical functions are ideal for this kind of work. A MATLAB programming simulation is used to test the suggested methods. To facilitate simulation, modelling, and analysis, we used MATLAB R2020b’s Robotics System Toolbox. We also created multi-domain structure using the graphic programming environment Simulink so that our proposed methodology could be compared with the latest methods. While numerous trials are employed to fine-tune the robots’ settings, multiple experiments on mobile robots are carried out to verify the proposed approach. Next, simulation tests validate the effects of the proposed method and its two main processes. In conclusion, several experiments are conducted to compare our recharge scheduling and selection procedures with the latest techniques in different scenarios.

###  Path-planning performance evaluation metrics

Selecting the optimal robot to complete the task will help to avoid delays or malfunctions. It’s also crucial to schedule robot recharging to manage and prevent failures. These are some of the most critical issues for the success of path planning as a system for mobile robotic networks. Evaluation criteria, such as Accuracy, path length, power consumption, and path smoothness, should be utilized to evaluate the system’s overall performance improvements. Table 5 displays an overview of the evaluation metrics that were used.

**Table 5:** Path-planning evaluation Metrics.

Metric	Definition
Accuracy (ACC.)	The ratio, which can be computed using Eq. (9), is the number of requests that are successfully provided to the total number of requests.
	$$\:\varvec{A}\varvec{c}\varvec{c}\:\varvec{\%}\:=\:\frac{\varvec{R}\varvec{d}}{\varvec{R}\varvec{t}}=\:\:\frac{\mathbf{R}\mathbf{d}}{\mathbf{R}\mathbf{d}+\mathbf{R}\mathbf{f}}\:$$(9)
	Rf: number of incorrect req, Rd: total of proper requests that the server that hosts the fog has successfully completed by delivering an item to a chosen robot, and Rt: the entire number of requests.
Path Length Percentage (PLP)	The total distance in meters that the robot takes to get from one place to another.
	$$\:\varvec{PLP}\:\varvec{\%}=\left(\:1-\frac{\varvec{A}\varvec{L}-\varvec{O}\varvec{L}}{\varvec{O}\varvec{L}}\:\right)\varvec{*}100\:\:\:\:\:\:\:\:\:\:\:\:\:\:\:\:\:\:\:\:\:\:\:\:\:\:\:$$(10)
	PLP represents the total length of the path percent, AL indicates the actual distance walked, and OL is the required optimum route length in meters, as shown by Eq. (10).
Power Consumption (PC)	The percentage of each robot’s charging capacity left after completing a series of requests and scheduling.
APT (Average Planning Time)	The average time spent planning a path is APT. The average amount of time an algorithm spends for planning is a critical metric since any algorithm used to design a path planning should be as fast as possible.
ART (Average Relative Time)	The average path planning time’s relative standard deviation. Equation (11) can be used to calculate the relative standard deviation of an algorithm’s average path planning time:
	$$\user2{ART}=\:\frac{\sqrt{\frac{1}{\varvec{N}.\varvec{K}}.\sum\:_{\varvec{i}=1}^{\varvec{N}.\varvec{K}}(\varvec{t}\varvec{i}-\varvec{A}\varvec{P}\varvec{T})2}}{\varvec{t}\varvec{m}}$$(11)
	Where N: destination points, ti is the planning time and K is the execution time of the algorithm
Path Smoothness (PS)	The path smoothness, or the amount of rotation the robot has to do to follow the path, indicates how smooth the path produced by the algorithm is calculated by Eq. (12) as mentioned in^28^.
	$$\user2{PS}=\:\frac{\sum\:_{\varvec{i}=1}^{\varvec{P}\varvec{a}\varvec{t}\varvec{h}\varvec{L}\varvec{e}\varvec{n}\varvec{g}\varvec{t}\varvec{h}}\varvec{\theta\:}\varvec{i}}{\left(\varvec{P}\varvec{a}\varvec{t}\varvec{h}\varvec{L}\varvec{e}\varvec{n}\varvec{g}\varvec{t}\varvec{h}-1\right)0.180^{\circ}}\:\in\:[0, 1]$$ (12)

### Implementing SP as a suggested method for robot selection

At this phase, the robot collection at the workspace is subjected to the recommended Selection Process (SP) in order to determine which robot is most suited for completing the task.

The suggested selection process is implemented centrally on the Fog server acting as the supervisor node. Each fog server is running the SP algorithm. The initial values of a request table are shown in Table 6.

**Table 6:** Request table (RT) initial values.

User	R_Priority	Code	Area	Weight (Wi)	Source Position	Destination Position
U_10	Critical	I(D-20)	Fog D	0.25 KG	30,2	15,83
U_2	Critical	I(A-3)	Fog A	3.5 KG	23, 66	89,50
U_50	Low	I(B-9)	Fog B	9 KG	9, 25	80,40
U_11	Low	I(A-93)	Fog C	2.8 KG	97,14	21,9
U_20	Low	I(C-33)	Fog C	6 KG	30,27	98,80
U_18	Normal	I(A-40)	Fog A	0.75 KG	50,70	7,70

The Python programming platform is used to apply the proposed RP (Rejection Process) and RA (Robot Assess), respectively, in accordance with our recommended criteria, as stated in algorithm1. Out of ten robots that are initially present in the workspace, a group of four robots is chosen. After that, they are assessed to identify which robot is most adept at finishing a particular task.

**Table 7:** Assess value (AV) for each Robot.

Robot No.	S	V	CHt (per min)	Distance from source	Distance to target	*P*	Wi	CHp	AV
R_1	1	10	0.5	2	3	8	5	70%	$\:0.83$
R_2	0	15	0.25	1	1	10	5	50%	0
R_3	1	20	0.25	4	5	7	5	75%	$\:1.49$
R_4	1	25	0.75	2	2	6	5	89%	$\:5.49$

Where P is the maximum payload, Wi is the item’s weight, S is the present state, v is velocity, CHt is the amount of time left to recharge the battery, and CHp is the robot’s charging percentage. Calculate an Assess Value (AV) for each of the four selected robots after using Eq. (1). Table 7 displays the Assess Value (AV) for every robot. Robot R4 was selected because it has the highest assessment value of 5.49.

$$\:AV\left(R1\right)=1*\frac{10+0.5}{2+3}*\left(\:\frac{8-3.5}{8}\text{*}\:70\text{\%}\:\right)=0.83$$

$$\:\:\:\:AV\left(R2\right)=0*\frac{15+0.25}{1+1}*\left(\:\frac{10-6}{10}\text{*}\:50\text{\%}\:\right)=0\:\:\:\:\:\:\:\:\:$$

$$\:\:\:\:\:\:\:\:\:AV\left(R3\right)=1*\frac{20+0.25}{4+5}*\left(\:\frac{7-0.78}{7}\text{*}\:75\text{\%}\:\right)=1.49\:\:\:\:\:\:\:\:\:$$

$$\:AV\left(R4\right)=1*\frac{25+0.75}{2+2}*\left(\:\frac{6-0.25}{6}\text{*}\:89\text{\%}\:\right)=5.49$$

### Experiments & comparative review of recent approaches with the proposed approach

**Fig. 7:**
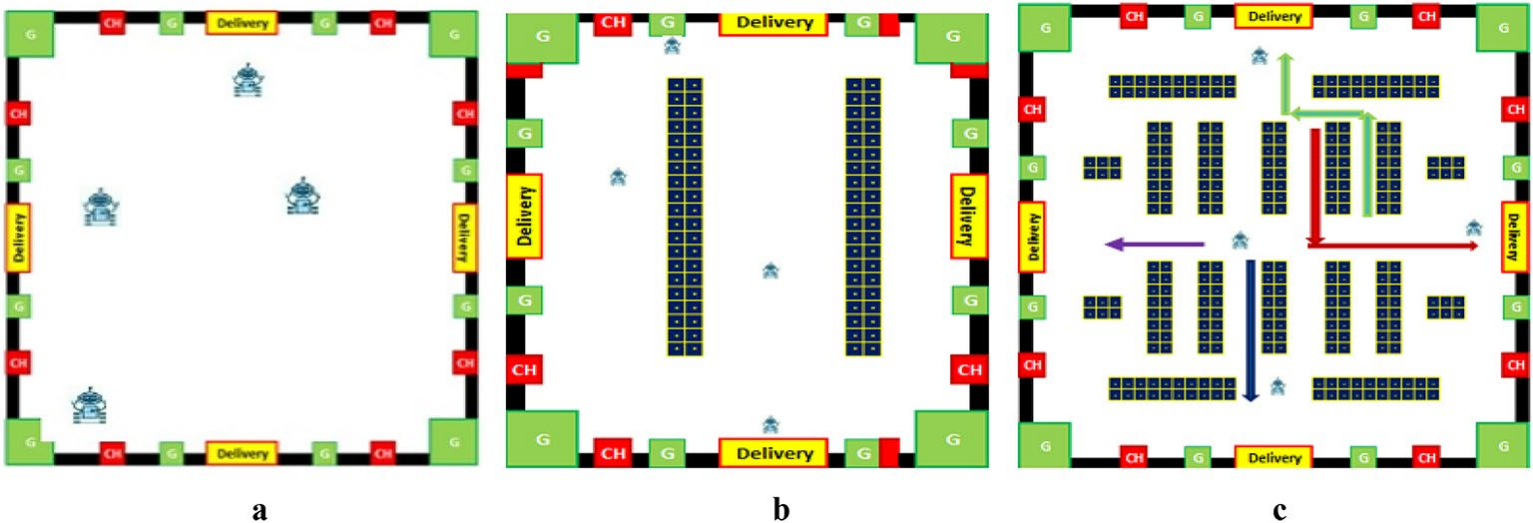
The simulation scenarios and the initial positions of the robots R1, R2, R3 and R4 at the start of the experiments.

This section assesses our proposed methodology’s applicability and efficiency compared to some recent algorithms. To this end, three distinct simulation workspaces have been defined as shown in Fig. 7. The workspaces’ grids plotted on the simulation scenarios are all the same size, 20*20, and four working robots, the robots selected from the SP algorithm, with the only variations being in the number and arrangement of obstacles. Figure 7.a illustrates how the first simulation scenario is done in a workspace map free of obstacles. Whereas, Fig. 7.b shows how the second simulation scenario established two blocks (such as potential barriers^14^). In the third simulation scenario, more complicated barriers are introduced, making it possible to analyze the algorithm’s effectiveness in handling blocks constrained from three sides, as seen in Fig. 7.c.

Tables 8 and 9, and 10 respectively show the parameters values of each of the selected four robots in the three mentioned scenarios after the SP and RSP are applied, based on each robot’s status, velocity, payload, and distance from the requested item, plus the Av values and the availability of each robot in each scenario.

**Table 8:**
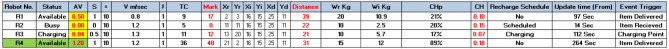
**Av** value and availability of each robot of the four robots in the 1^st^ scenario.

**Table 9:**
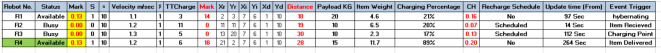
**Av **value and availability of each robot of the four robots in the 2nd scenario.

**Table 10:**
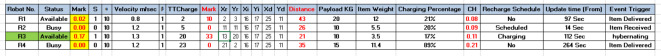
**Av **value and availability of each robot of the four robots in the 3rd scenario.

**Fig. 8:**
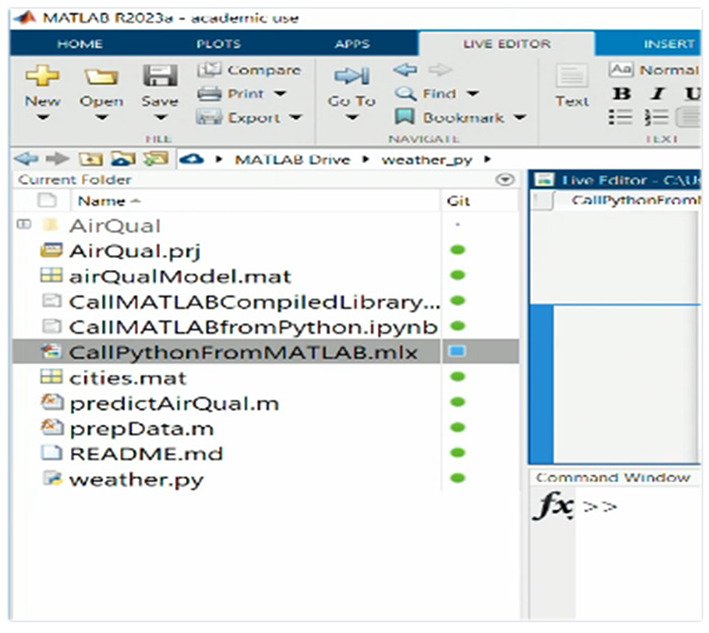
Deploying Python code to MATLAB.

Figure 8 shows how to generate Python code for the suggested algorithms SP and RSP and then use MATLAB to get in connection with the MATLAB engine API for Python. To identify which robots are best suited to respond to a particular request, the fog server uses both the SP algorithm and the fuzzy technique of recharge scheduling the robots. The recommended algorithm then determines which robot is the best by assigning an Assess Value to each robot. When our methods’ Python code was incorporated within the MATLAB platform to simulate our approach for each scenario.

**Table 11:** Average evaluation metrics values for the proposed approach.

Set	Req	Acc.	PLP	PS	PC	APT	ART
Set-1	250	99.6%	96.0%	0.87	99.6%	3.1	13.3
Set-2	500	99.4%	96.0%	0.90	99.4%	3.2	21.9
Set-3	750	99.3%	96.0%	0.34	99.3%	3.3	33.2
Set-4	1000	99.2%	97.0%	0.55	99.2%	3.6	45.6
Set-5	1250	99.2%	96.8%	0.68	99.2%	3.9	59.0
Set-6	1500	99.3%	96.7%	0.75	99.3%	4.2	75.4
Set-7	1750	99.3%	96.6%	0.64	99.3%	4.3	94.2
Set-8	2000	99.4%	96.5%	0.89	99.4%	4.7	119.5
AVERAGE	-	99.3%	96.4%	0.70	99.3%	3.8	57.8

**Fig. 9:**
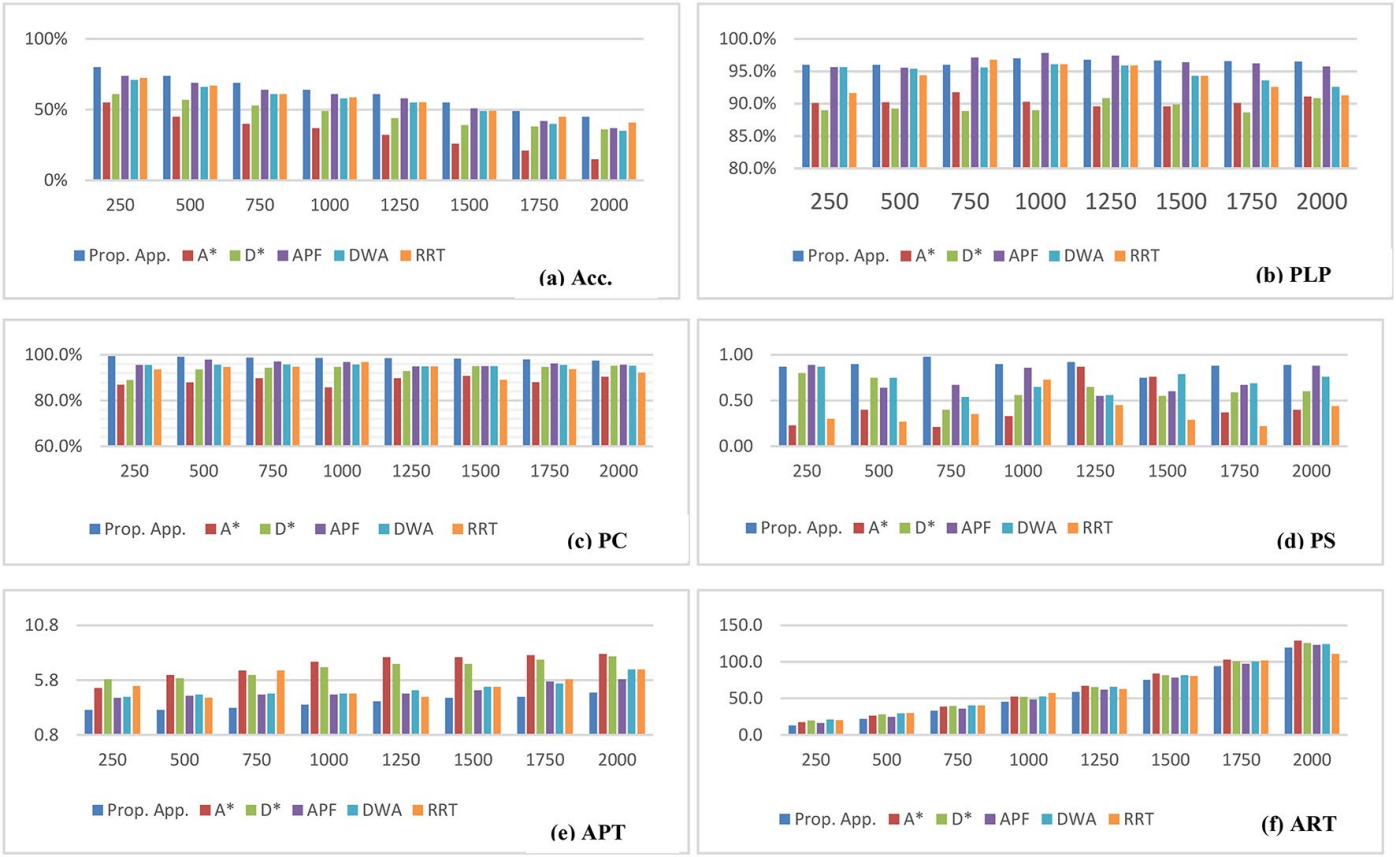
Average Simulation Results of the Evaluation Metrics of our proposed approach compared to recent methods.

Table 11 Shows the performance evaluation metrics for our suggested technique. Each of the eight sets of randomly selected requests, which had 250 requests total and were added one at a time to the previous set, has its performance evaluation percentages displayed in Table 11. The simulation results show that our proposed method has improved the accuracy and power consumption of recent planning techniques, as evidenced by the charts of Fig. 9.A, 9.B, 9.c, 9.D, 9.E, and 9.F, respectively, and Table 12. Regarding outcome percentages and values, the proposed method outperforms earlier algorithms examined in our trials (A*, D*, APF, DWA, and RRT) if applied to the same dynamic scenarios.

**Table 12:** Simulation Results.

Methodology	Prop. App.	A^*^[14]	D^*^ [17]	DWA[19]	APF[21]	RRT [18]
Year	2024	2019	2020	2022	2023	2023
ACC.	98.6%	87.7%	93.8%	95.5%	96.2%	93.7%
PLP	96.4%	90.4%	89.5%	94.9%	96.5%	94.1%
PC	62.1%	34.0%	47.0%	54.0%	59.7%	56.0%
PS	0.886	0.446	0.613	0.701	0.720	0.381
APT	3.8	4.5	7.0	4.9	4.7	5.4
ART	58.0	65.0	64.0	65.0	61.0	63.0

**Fig. 10:**
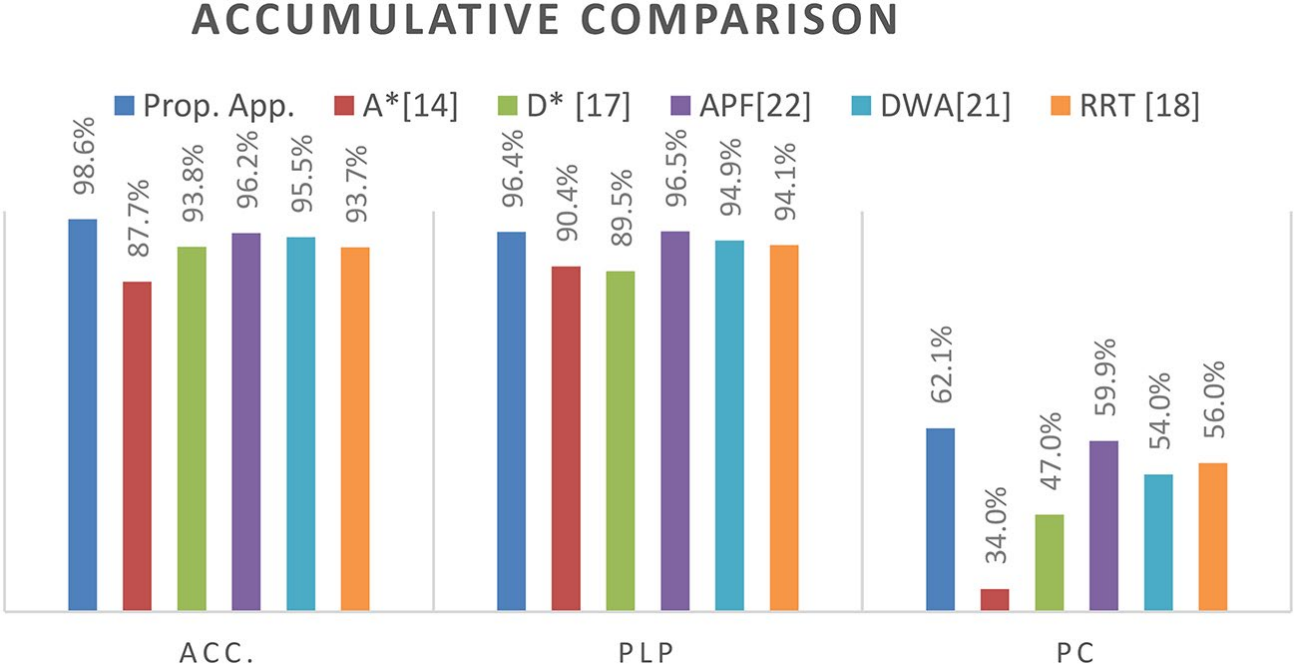
Average Accumulative Comparison of our proposed approach to recent methods.

In terms of Accuracy, Path Length Percentage, and Power Consumption, Fig. 10 presents an accumulative comparison of our proposed approach to recent algorithms. It shows that the proposed approach offered the best average accuracy, with an average of 98.6%, PLP average percentage of 96.4%, and PC average percentage of 62.1%.

## Conclusion and future work

This paper suggests a new approach for determining which robots are most suited for a certain task, eliminating unsuitable robots, and scheduling robots that require recharging. This improves the accuracy and energy efficiency of real-time path planning methods. The server node manages processing and computations, saves energy, and expedites the system’s computation time, which decreases the burden upon the mobile robots’ capabilities. The analysis showed that the suggested methodology outperformed several existing path-planning strategies, with the shortest time for processing and an estimated accuracy rating of 98.6%.

Enhancing the path planning method of the system to identify other routes for every robot is one of our future goals. This is because it’s possible that any unforeseen or spontaneous modifications made within the workspace could have an impact on the fog server’s global database. We also propose to combine multitask planning with deep robot learning models. This can increase the low latency of multi-agent systems.

## Data Availability

All data generated or analyzed during this study are included in this published article, and any further information regarding the current study is available from the corresponding author on reasonable request.
